# The survival outcome and complication of secondary cytoreductive surgery plus chemotherapy in recurrent ovarian cancer: a systematic review and meta-analysis

**DOI:** 10.1186/s13048-021-00842-9

**Published:** 2021-07-13

**Authors:** Ting Ding, Dan Tang, Mingrong Xi

**Affiliations:** 1 Department of Obstetrics and Gynecology, West China Second Hospital, Key Laboratory of Birth Defects and Related Diseases of Women and Children, Sichuan University, Ministry of Education, Number 20, 3rd Section, South Renmin Road, Chengdu, 610041 Sichuan Province China; 2 Department of Obstetrics and Gynecology, West China Second University Hospital, Key Laboratory of Birth Defects and Related Diseases of Women and Children, Sichuan University, Ministry of Education, Number 20, 3rd Section, South Renmin Road, Chengdu, 610041 Sichuan Province China

**Keywords:** Ovarian cancer, Secondary cytoreductive surgery, Meta-analysis, Systematic review

## Abstract

**Objective:**

The aim of this meta-analysis was to assess the effectiveness and safety of secondary cytoreductive surgery plus chemotherapy (SCS + CT) in recurrent ovarian cancer (ROC). Our secondary purpose was to analyze whether patients could benefit from complete resection.

**Methods:**

We searched EMBASE, MEDLINE, the Cochrane Database of Systematic Reviews and Cochrane Central Register of Controlled Trials, from inception to April 2021. We used appropriate scales to assess the risk of bias. Data from included studies that reported median PFS or OS were weighted by individual study sample size, and aggregated for meta-analysis. We calculated the pooled proportion of complications within 30 days after surgery.

**Results:**

We identified 13 articles, including three RCTs and ten retrospective cohort studies. A total of 4572 patients were included, of which 916 patients achieved complete resection, and all patients were comparable at baseline. Compared with chemotherapy alone, SCS + CT significantly improved the PFS (HR = 0.54, 95% CI: 0.43–0.67) and OS (HR = 0.60, 95% CI: 0.44–0.81). Contrary to the results of cohort studies, the meta-analysis of RCTs showed that SCS + CT could not bring OS benefits (HR = 0.93, 95% CI: 0.66–1.3). The subgroup analysis showed the prognostic importance of complete resection. Compared with chemotherapy alone, complete resection was associated with longer PFS (HR = 0.53, 95% CI: 0.45–0.61) and OS (HR = 0.56, 95% CI: 0.39–0.81), while incomplete resection had no survival benefit. Additionally, complete resection could maximize survival benefit compared with incomplete resection (HR = 0.56, 95% CI: 0.46–0.69; HR = 0.61, 95% CI: 0.50–0.75). The pooled proportion for complications at 30 days was 21% (95% CI: 0.12–0.30), and there was no statistical difference in chemotherapy toxicity between the two groups.

**Conclusion:**

The review indicated that SCS + CT based regimens was correlated with better clinical prognosis for patients with recurrent ovarian cancer, but the interpretation of OS should be cautious. The meta-analysis emphasizes the importance of complete resection, suggesting that the potential benefits of prolonging survival may outweigh the disadvantages of any short-term complications associated with surgery.

**Supplementary Information:**

The online version contains supplementary material available at 10.1186/s13048-021-00842-9.

## Highlights

- There is significant heterogeneity in literature with the use of SCS + CT in recurrent ovarian cancer, and one prospective evidence discourages SCS + CT.
- The meta-analysis based on the latest evidence of randomized controlled trials and other related publications.
- Compared with chemotherapy alone, SCS + CT is associated with longer PFS, but the interpretation of OS should be cautious.
- Complete resection is shown to maximize survival benefit compared with incomplete resection and chemotherapy alone.
- The incidence of complications was low within 30 days after receiving surgery and there was no statistical difference in chemotherapy toxicity between the two groups.

## Introduction

After first-line chemotherapy and targeted maintenance therapy, about 80% of patients with advanced ovarian cancer will relapse [1]. For patients with recurrent ovarian cancer (ROC), secondary cytoreductive surgery plus chemotherapy (SCS + CT) is a widely practiced option [2]. SCS is defined as surgery to further debulk the recurrent tumour after completing the main treatment and a certain period of remission [3]. However, the role of SCS + CT in patients with ROC has not been defined by level-1 evidence. Among ROC patients, the difference in progression-free survival (PFS) and overall survival (OS) associated with SCS + CT compared with chemotherapy alone is still unclear.

In 2006, an exploratory study (DESKTOP I) identified factors associated with improved survival after SCS + CT, with data from 25 member institutions [4]. DESKTOP II applied three criteria (PS 0, complete resection at initial surgery, and ascites < 500 ml), naming them as “positive AGO-score” to prospectively select patients who might benefit from SCS + CT. In total, 76% of the patients with a positive AGO score achieved an optimal cytoreduction [5].

Subsequently, four randomized controlled trials (RCTs) were conducted to compare SCS + CT with chemotherapy alone in ROC patients: GOG-0213 (ClinicalTrials.gov number NCT00565851), DESKTOP III (ClinicalTrials.gov number NCT01166737), SOCceR (Netherlands Trial Register number NL3137) and SOC-1 (ClinicalTrials.gov number NCT01611766) [6]. The SOCceR trial was terminated early due to poor enrollment [7].

GOG-0213 trial randomized patients with ROC to SCS + CT or chemotherapy alone, and nearly 80% of patients in each group received bevacizumab [8]. In published data, there was no improvement in PFS and OS in surgery group, and even complete resection did not affect the prognosis of patients [9]. These findings changed the previous understanding of the significance of SCS + CT for platinum-sensitive ROC. The DESKTOP III trial enrolled a total of 407 patients who relapsed ≥ 6 months after platinum chemotherapy with a positive AGO score [10]. The final analysis demonstrated that SCS + CT significantly improved the PFS and OS [11]. SOC-1 trial enrolled 357 patients with platinum-sensitive relapsed ovarian cancer with a platinum-free interval of at least 6 months after the end of first-line chemotherapy and were predicted to have potentially resectable disease according to the recurrence score (PET-CT imaging and iMODEL score) [12]. The results showed that SCS + CT was associated with longer PFS but not beneficial to OS.

One published meta-analysis suggested that SCS + CT significantly improved PFS for patients with ROC, but did not improve OS [13]. However, the study did not provide enough available data and did not assess the complications after surgery, making it difficult to fully estimate the clinical benefit of SCS + CT.

Given the conflicting evidence regarding the role of SCS + CT, the study aimed to add additional data by including RCTs and a number of recent publications. The purpose of this meta-analysis was to evaluate the prognostic impact of SCS + CT on patients with ROC. Our secondary purpose was to analyze whether patients could benefit from complete resection. Considering the potential role of unmeasurable confounding factors in the selection of patients, we also performed a sensitivity analysis to determine the impact of unmeasurable confounding factors on our results.

## Method

### Data sources and searches

This analysis was reported in accordance with the Preferred Reporting Items for Systematic Reviews and Meta-Analyses (PRISMA) guidelines. The protocol was registered to the International Prospective Register of Systematic Review (PROSPERO) with registration number CRD42020209013. From 1946 to April 2021, the following databases were systematically searched: EMBASE, MEDLINE, the Cochrane Database of Systematic Reviews and Cochrane Central Register of Controlled Trials. We also searched abstracts of scientific meetings, registers of clinical trials (https://www.clinicaltrials.gov/) and reference lists of included studies. The search terms including "ovarian cancer", and “secondary cytoreductive surgery”. Manually filter the citation list of the retrieved articles to ensure the sensitivity of the search strategy.

### Study selection

Articles were accepted if they complied with the following eligibility criteria: 1) Clinical trials, cohort studies or case–control studies; 2) Patients with a diagnosis of recurrent ovarian cancer; 3) Comparison of secondary cytoreductive surgery plus chemotherapy and chemotherapy alone; 4) Median PFS, OS, the rate of complete resection and complications were reported; 5) Full-text publication; 6) English Publication. All titles identified by the search have been evaluated, and all potentially relevant publications have been fully searched. Two review authors (KG, TA) independently evaluated the eligibility of the papers and resolved their disagreements by discussion or by appeal to a third review author (AB).

### Data extraction

The following detailed information was extracted from the included studies according to pre-designed criteria: name of the first author, year of publication, journal, study design, study population, median or average age, intervention method, median OS, median PFS, complications and toxicity. We extracted the hazard ratio (HR) and 95% confidence interval (CI) of PFS and OS for each article. The score of completeness of cytoreduction was evaluated according to Sugarbaker [14]. R0: complete resection (no residual disease); R1: the residual disease with nodules measuring less than 2.5 mm; R2: the residual disease with nodules measuring between 2.5 mm and 2.5 cm; R3: the residual nodules greater than 2.5 cm. When possible, all the data extracted were relevant to an intention-to-treat (ITT) analysis. Two review authors (KG, TA) independently extracted the data into a data abstraction form specially designed for the review. When necessary, the reviewer resolved the differences of opinion by discussing or appealing to a third review author (AB).

### Statistical analysis

We used Review Manager 5.3 (http://www.cochrane.org) and Stata 15.0 software for statistical analysis. A χ2 heterogeneity test and sensitivity analysis were performed to assess the existence of statistical heterogeneity between studies. If the I2 > 50%, the random-effects model was applied, otherwise the fixed-effects model was adopted [15]. The forest plots showed graphical representation of each study and pooled analysis. The weight provided by each study in the meta-analysis was reported graphically as a square of different size. The confidence interval (CI) was expressed as a horizontal line passing through the square for each study. The pooled HR was represented as a lozenge in the forest plot, and the size corresponded to the 95% CI of the HR. A p value ≤ 0.05 was considered significant.

### Risk of bias assessment

We assessed the methodological quality in cohort studies using the Newcastle Ottawa Scale (NOS) and Minors Scale. Studies were classified as low, moderate or high risk of bias based on its overall score. The Cochrane Collaboration's Risk of Bias Tool and Jadad Scale were used for randomized controlled trials. To investigate publication bias, we performed a funnel plot analysis.

## Results

### Search results and Study quality

The literature search identified 3325 articles and evaluated the eligibility of 37 articles. We excluded 24 studies, 17 articles had no control group [4, 16–31], three articles were conference abstracts, two articles did not meet the inclusion and exclusion criteria [32, 33], one article received radiotherapy in the control group [34], and one prospective trial terminated early due to poor enrollment [35].

**Fig. 1:**
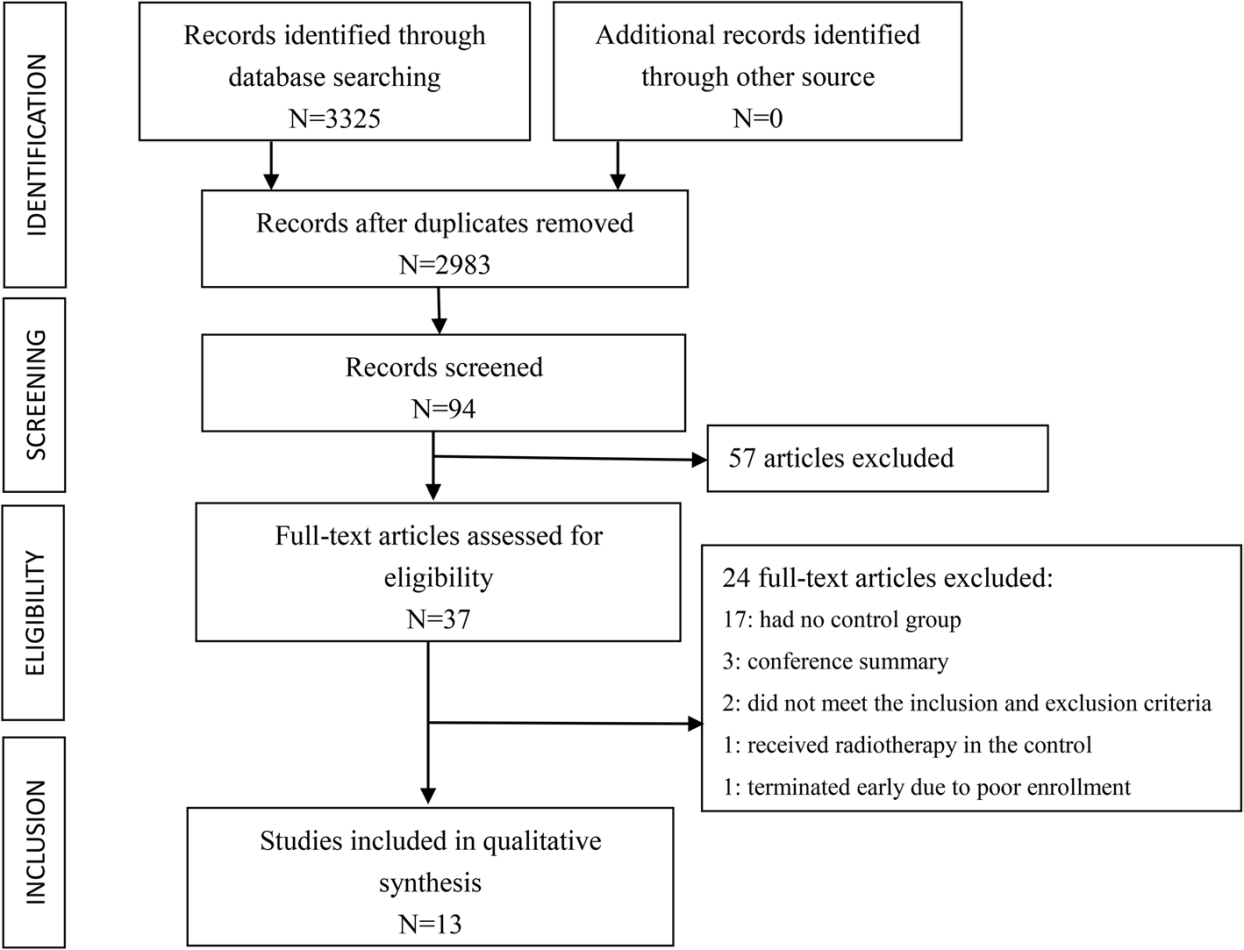
Flow diagram of study selection for systematic review

Finally, 13 articles were identified, including three RCTs and ten retrospective cohort studies [9, 11, 12, 36–45]. Nine of those 13 articles reported complications with 30 days after surgery and were eligible for quantitative synthesis. Three studies reported on the 30-day reoperation rate. The PRISMA flow chart summarizing the process of study selection is shown in Fig. 1.

In total, the number of patients included in the meta-analysis was 4572, ranging from 52 to 964 patients per study. Of the 4572 patients, 1446 (31.6%) received SCS + CT and 3126 (68.4%) received chemotherapy alone. All articles were published between 2005 and 2021, with patients included from 1985 to 2019 (Table 1).

**Table 1:** Characteristics of included studies

**Study**	**Year**	**Study type**	**Total** **n**	**HR of PFS** **(95% CI)**	**P value**	**HR of OS** **(95% CI)**	**P value**	**Jadad Score**
Coleman et al [9]	2019	RCT (GOG-0213)	485	0.82 (0.66–1.01)	P = 0.073	1.29(0.97–1.72)	P > 0.05	5
du Bois et al. ^a^ [11]	2020	RCT (DESKTOP III)	407	0.66 (0.52–0.83)	P < 0.001	0.76 (0.59–0.97)	P < 0.05	5
Shi et al [12]	2021	RCT (SOC-1)	357	0.58 (0.45–0.74)	P < 0.001	0.82 ( 0.57–1.19)	P = 0.29	5
**Study**	**Year**	**Study type**	**Total** **n**	**HR of PFS (95% CI)**	**P value**	**HR of OS** **(95% CI)**	**P value**	**Minors Score**
Gockley et al [36]	2019	Retrospective	626	NR	NR	0.45(0.32–0.65)	P < 0.001	20
Felsinger et al [37]	2018	Retrospective	62	NR	P = 0.01	NR	P = 0.007	18
Szczesny et al [38]	2018	Retrospective	397	0.45 (0.32–0.62)	P < 0.001	0.5 (0.32–0.70)	P < 0.001	18
Lee et al [39]	2015	Retrospective	964	0.42 (0.33–0.52)	P < 0.001	0.49 (0.39–0.61)	P < 0.001	20
Ortega et al [40]	2020	Retrospective	71	0.28(0.15–0.5)	p = 0.001	0.33(0.17–0.6)	P = 0.001	20
So M et al [41]	2019	Retrospective	52	0.45(0.22–0.91)	P = 0.027	0.28(0.11–0.72)	P = 0.008	20
Güngör et al [42]	2005	Retrospective	75	NR	NR	NR	P = 0.03	17
Oksefjell et al [43]	2009	Retrospective	789	NR	NR	NR	P < 0.01	16
Takahashi et al [44]	2017	Retrospective	112	0.57(0.33–0.97)	P = 0.02	0.66(0.33–1.31)	P = 0.23	19
Kajiyama et al [45]	2019	Retrospective	169	NR	P = 0.114	NR	P = 0.32	17

The number, HR, 95%CI or P value is shown for SCS + CT versus CT. ^a^study including latest data presented at ASCO 2020

*Abbreviations*: *HR* hazard ratio, *95%CI* 95% confidence interval, *PFS* progression-free survival, *OS* overall survival, *NR* not reported

This study analyzed whether the included patients were comparable at baseline (Additional file 1: Table S1). Eastern Cooperative Oncology Group (ECOG) performance status (PS) was reported in four studies, and there was no significant difference in PS 0 between the two groups (65.9% versus 68.7%; P = 0.32) (Additional file 1: Figure S1). The initial diagnosis of FIGO staging was reported in ten studies, and there was a significant difference between the two groups (P = 0.0001). 686 (71.8%) patients in the SCS + CT group had stage III or IV disease compared to 2158 (81.5%) patients in the chemotherapy group. Tumor histology was reported in eight studies, of which 1790 (77%) patients had high grade serous carcinoma, and there was no statistical difference between the two groups (77.4% versus 76.7%; P = 0.43). Four studies reported the presence or absence of ascites at the time of relapse, and there was no statistical difference between the two groups (87.1% versus 70.5%; P = 0.44). Residual disease after initial operation was reported in five articles, and there was no statistical difference between the two groups (53.6% versus 52.9%; P = 0.24). Seven articles reported the number of recurrent lesions by imaging. The number of recurrent lesions in the chemotherapy group was significantly higher than that in the surgery group (49.2% versus 23.4%; P < 0.0001). Five articles reported the site of recurrent lesions, and there was no statistical difference between intra-abdominal lesions and extra-abdominal lesions (82.6% versus 89.3%; P = 0.48).

Included patients in each study were divided into experiment group (SCS + CT) and control group (CT), and the control group received platinum-based chemotherapy. The chemotherapy regimen used in each study was the standard treatment regimen during the study period. None of the included studies used high temperature intraperitoneal chemotherapy (HIPEC).

### Secondary cytoreductive surgery plus chemotherapy vs. Chemotherapy alone

**Fig. 2:**
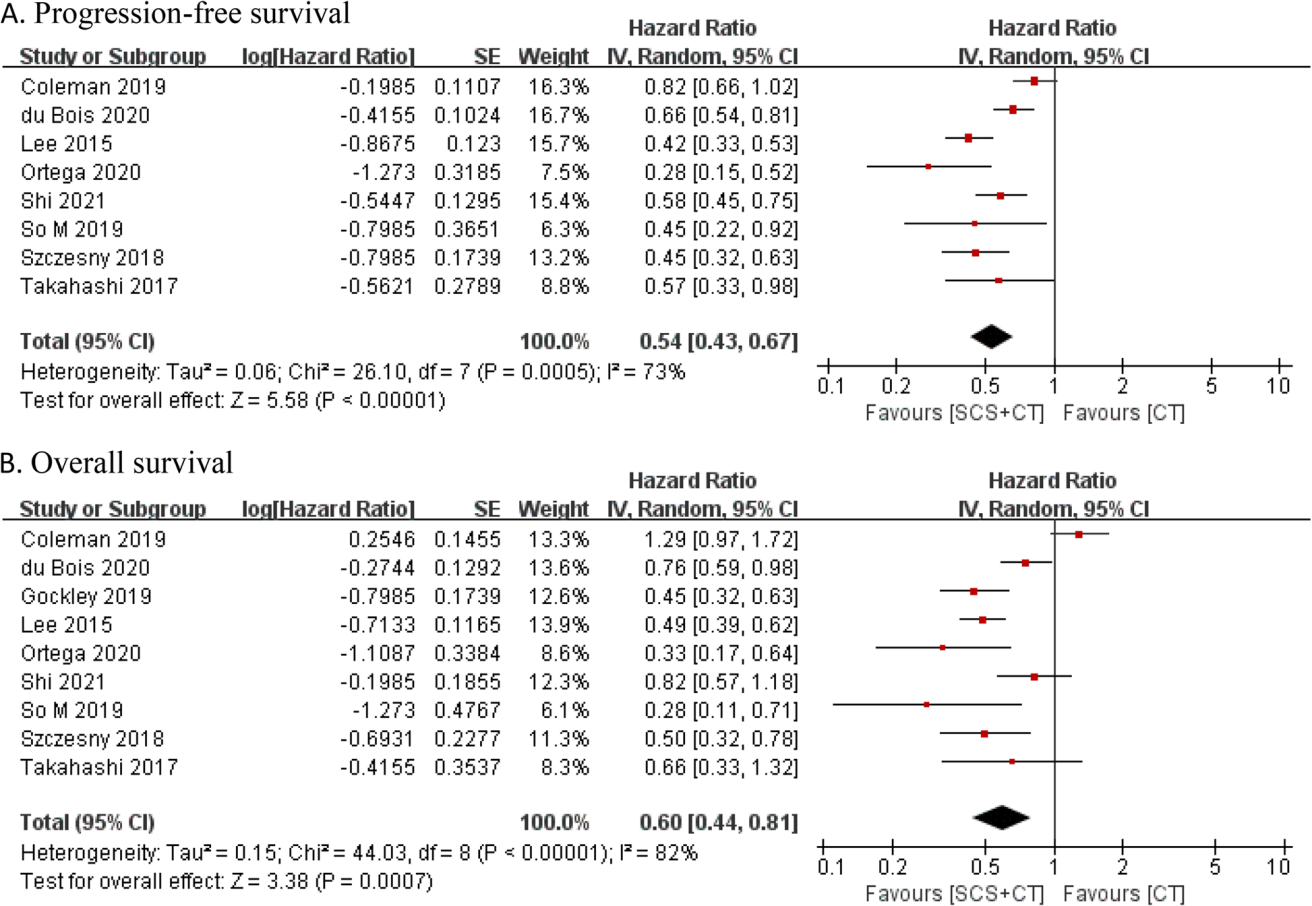
Forest plots for comparison SCS + CT versus CT in recurrent ovarian cancer. Abbreviations: SCS, secondary cytoreductive surger; CT, chemotherapy

All included studies compared the efficacy of SCS + CT and chemotherapy alone in ROC patients. Four cohort studies did not report standard errors or 95% confidence intervals of log HR, only the significance probabilities of significant variables in the Cox model were reported. Nine studies were eligible to assess the impact of SCS + CT on OS, and eight studies provided available data to calculate the HR of the PFS. Pooled data demonstrated that compared with chemotherapy alone, the SCS + CT significantly improved the PFS (HR = 0.54, 95% CI: 0.43–0.67, P < 0.00001) and OS (HR = 0.60, 95% CI: 0.44–0.81, P = 0.0007) (Fig. 2).

Due to the contradictions between the results of RCTs, we conducted a subgroup analysis (Additional file 1: Figure S2). The subgroup analysis indicated that SCS + CT did not improve OS (I2 = 75%; HR = 0.93, 95% CI: 0.66–1.31, P = 0.69), which was contrary to the results of cohort studies (I2 = 0%; HR = 0.47, 95% CI: 0.40–0.55, P < 0.00001).

The heterogeneity test was conducted based on the HR and 95% CI of PFS (I^2^ = 73%, P = 0.0005) and OS (I^2^ = 82%, P < 0.001). The results showed that the nine studies were highly heterogeneous. To investigate the impact of the individual study on the pooled data, we conducted a sensitivity analysis (Additional file 1: Figure S3). Every study was deleted consecutively to test the stability of the data. The result of PFS and OS was robust, sequential omission of data from any individual study did not affect the results.

### Subgroup analysis

We conducted a subgroup analysis to assess the impact of complete resection on survival outcomes. All included articles reported the number of patients with complete resection (Table 2). Of the 1446 patients who received SCS + CT, 916 patients achieved complete resection.

**Table 2:** Characteristics of patients with complete resection included in the study

Author	SCS n	CT n	R0 n	R0 vs. R1 + R2 + R3		R0 vs. CT		R1 + R2 + R3 vs. CT	
				PFS	OS	PFS	OS	PFS	OS
Coleman et al [9]	240	245	150	0.51 (0.36–0.71)	0.61 (0.40–0.93)	0.62(0.48–0.80)	1.03(0.74–1.46)	NR	NR
du Bois et al [11]	206	201	138	NR	NR	0.56(0.43–0.73)	0.57(0.43–0.76)	NR	NR
Shi et al [12]	182	175	132	NR	NR	0.5(0.37–0.66)	0.59(0.38–0.91)	0.91(0.61–1.36)	1.79(1.07–2.99)
Gockley et al [36]	146	480	62	NR	NR	NR	0.38(0.23–0.64)	NR	0.8(0.62–1.03)
Felsinger et al [37]	30	32	24	NR	NR	NR	NR	NR	NR
Szczesny et al [38]	75	322	60	NR	NR	0.34(0.23–0.51)	0.36( 0.22–0.57)	NR	NR
Lee et al [39]	187	777	140	0.59(0.46- 0.76)	0.61(0.48- 0.79)	NR	NR	NR	NR
Ortega et al [40]	37	34	33	NR	NR	NR	NR	NR	NR
So M et al [41]	22	30	16	NR	NR	NR	NR	NR	NR
Güngör et al [42]	44	31	34	NR	NR	NR	NR	NR	NR
Oksefjell et al [43]	217	572	76	NR	NR	NR	NR	NR	NR
Takahashi et al [44]	35	77	33	NR	NR	NR	NR	NR	NR
Kajiyama et al [45]	25	144	18	NR	NR	NR	NR	NR	NR

*Abbreviations*: *SCS* secondary cytoreductive surgery, *CT* chemotherapy, *R0* complete resection (the presence of zero macroscopic residuum), *R1* + *R2* + *R3* incomplete resection, *PFS* progression-free survival, *OS* overall survival, *NR* not reported

**Fig. 3:**
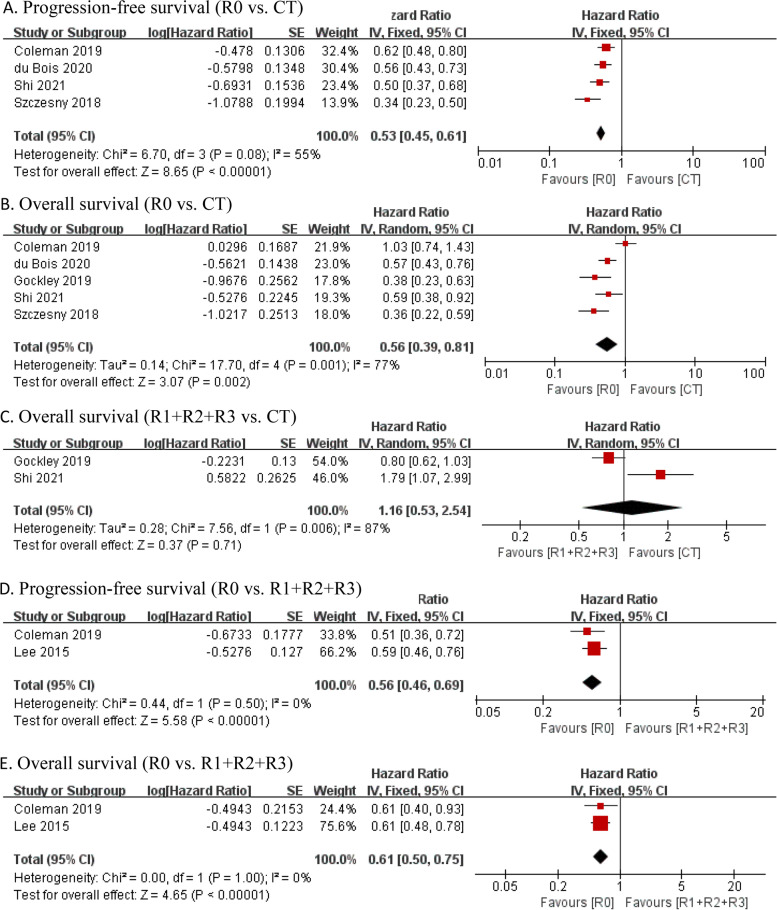
Forest plot for subgroup analysis of recurrent ovarian cancer. **A**: PFS of complete resection versus chemotherapy alone; **B**: OS of complete resection versus chemotherapy alone; **C**: OS of incomplete resection versus chemotherapy alone; **D**: PFS of complete resection versus incomplete resection; **E**: OS of complete resection versus incomplete resection. Abbreviations: SCS, secondary cytoreductive surger; CT, chemotherapy; R0: complete resection; R1 + R2 + R3: imcomplete resection

Six studies were eligible to compare the effects of complete resection and chemotherapy alone, but one of them did not report the standard error or 95% CI of log HR. The meta-analysis of five articles evaluated 1971 patients (complete resection group: 542, chemotherapy alone group: 1429). Pooled data demonstrated that compared with chemotherapy alone, complete resection significantly improved PFS (HR = 0.53, 95% CI: 0.45–0.61, P < 0.00001) and OS (HR = 0.56, 95% CI: 0.39–0.81, P = 0.002) (Fig. 3).

In addition, two articles compared the efficacy of incomplete resection and chemotherapy alone, and evaluated 855 patients (incomplete resection group: 194; chemotherapy alone group: 661). The results indicated that compared with chemotherapy alone, incomplete resection did not improve OS (HR = 1.16, 95% CI: 0.53–2.54, P = 0.71). Only the SOC-1 trial reported PFS, 12.6 months in the incomplete resection group and 11.9 months in the chemotherapy group (HR = 0.91, 95% CI: 0.61–1.36).

In order to explore the impact of residual tumors on survival outcomes, we compared the effects of complete resection and incomplete resection on the prognosis of patients. Two articles were included in the meta-analysis, and evaluated 427 patients (complete resection group: 290; incomplete resection group: 137).The results showed that complete resection were associated with longer PFS (HR = 0.56, 95% CI: 0.46–0.69, P < 0.00001) and OS (HR = 0.61, 95% CI: 0.50–0.75, P < 0.00001).

The results of heterogeneity test showed that the six articles were highly heterogeneous (R0 vs. CT: I2 = 77%, P = 0.001; R1 + R2 + R3 vs. CT: I2 = 87%, P = 0.006). Sensitivity analysis indicated that the result of PFS and OS was reliable, and sequential omission of data from any individual study did not affect the results (Additional file 1: Figure S4).

### Complications and toxicity

**Fig. 4:**
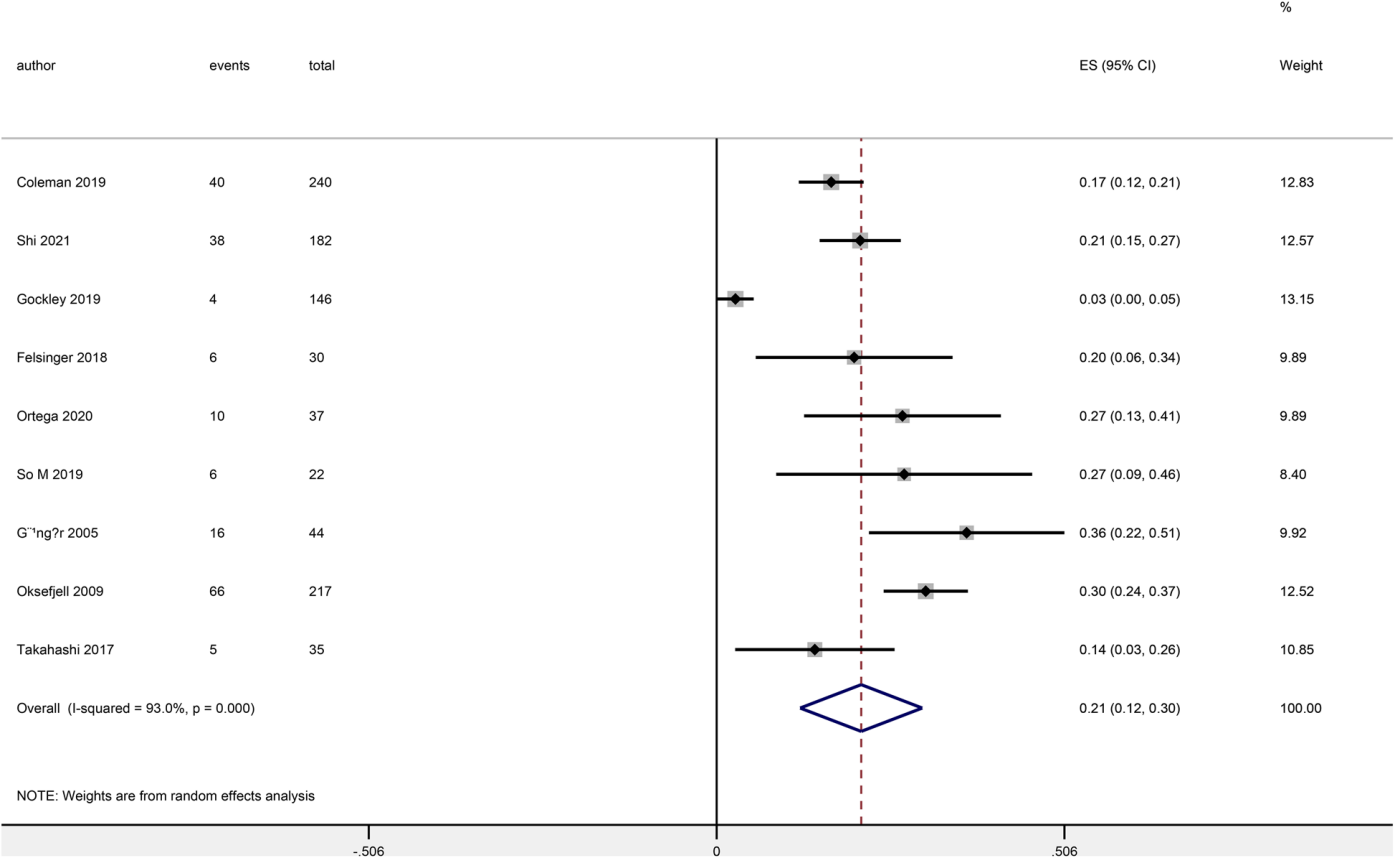
The pooled proportions for 30-day complications among recurrent ovarian cancer patients after secondary cytoreductive surgery

Nine articles reported that 347 patients underwent bowel resection, and two articles reported that 193 patients suffered haemorrhage or required blood transfusion during surgery (Additional File 1: Table S2). Nine articles reported the incidence of complications about intraoperative or postoperative. 191 (20.1%) of 953 patients had complications within 30 days after SCS, and the pooled proportion among patients was 21% (95% CI: 0.12–0.30) (Fig. 4). 8 (4.1%) patients had pleural effusion, 86 (45.0%) patients had intestinal obstruction, 7 (3.7%) patients had deep vein thrombosis, and 25 (13.1%) patients had repeated operations. In addition, 27 (14.1%) patients developed postoperative infections, including wound infections, abdominal infections, lung infections and urinary tract infections. 4 (2.1%) patients developed heart events, and 34 (17.9%) patients had other complications (bleeding, intestinal fistula and renal impairment). ​Most patients had grade 1–2 complications, and only the GOG-0213 trial reported one patient died within 30 days after surgery due to pulmonary embolism [9].

Only two articles (SOC-1 and GOG-0213 trial) reported the toxicity of chemotherapy, and there was no statistical difference between the two groups (P = 0.90) [9, 12]. In SOC-1 trial, 41 (23%) of 182 patients in the SCS + CT group and 31 (18%) of 175 in the chemotherapy group had grade 3 or worse adverse events during chemotherapy (P > 0.2). The most common grade 3–4 adverse events during chemotherapy were neutropenia (SCS + CT group: 29; CT group: 19), leucopenia (SCS + CT group: 14; CT group: 8), and anaemia (SCS + CT group: 10; CT group: 9) (P > 0.5). Two patients in the SCS + CT group discontinued treatment due to chemotherapy-related toxicity. In GOG-0213 trial, 183 (76.3%) of 240 patients in the SCS + CT group and 194 (79.2%) of 245 in the chemotherapy group had grade 3 or worse adverse events during chemotherapy (P = 0.44). Two patients in the chemotherapy group died due to cardiac events.

### Risk of bias in included studies

We assessed all full-text studies for methodological quality (Additional file 1: Appendix 2–3-4–5). In all included studies, appropriate statistical techniques were used to analyze PFS and OS, and multivariate analysis was used to adjust for important prognostic factors in the Cox regression model, making the two groups comparable. In addition, all included studies appeared to include a representative sample of patients with ROC that had been cytoreduced via SCS. We included only sufficiently large studies and used multivariate analysis to control various co-factors to reduce the possibility of selection bias. Considering the fact that the comparison of residual diseases almost came from cohort studies, we should be cautious when conducting data extraction and meta-analysis. In short, although cohort studies had a high risk of bias, each study was representative and comparable.

Begg’s test and Egger’s test were applied to evaluate the bias of publication, and there was no significant bias in PFS (PB = 0.266, PE = 0.226) and OS (PB = 0.754, PE = 0.433) (Additional file 1: Figure S5).

## Discussion

Whether SCS + CT can improve the prognosis of ROC and whether patients can benefit from complete resection are still clinically controversial issues. Almost all retrospective studies indicated that SCS + CT was beneficial to patients with ROC, especially those who had achieved complete resection. However, the results of RCTs in the past 10 years were contrary to this, and the results of several RCT trials were contradictory [9, 11, 12]. The results of GOG-0213 trial showed that there was no significant difference of PFS (18.9 vs. 16.2 months; P = 0.073) and OS (50.6 vs. 64.7 months; P = 0.08) between the SCS + CT group and the chemotherapy group [9]. Even if complete resection was achieved, it did not affect the prognosis of the patient (56.0 vs.64.7 months; P > 0.05). Primary endpoint analysis of DESKTOP III trial showed that median OS of 53.7 months with and 46.2 months without surgery (P = 0.03); median PFS was 18.4 and 14 months (P < 0.001) [11]. In SOC-1 trial, the PFS of SCS + CT group and chemotherapy group was 17.4 months and 11.9 months, respectively (P < 0.0001), and the OS was 58.1 months and 53.9 months, respectively (P > 0.05) [12].

In this meta-analysis, three RCTs and ten cohort studies were included. Pooled data demonstrated that SCS + CT significantly improved the PFS (P < 0.0001) and OS (P = 0.02) in patients with ROC. Subgroup analysis of RCTs indicated that SCS + CT had no effect on OS (P = 0.67), which was contrary to the results of cohort studies (P < 0.0001).

In order to explain the contradictions between the research results, several key points need to be further discussed. First, adjuvant therapy might affect the efficacy of surgery. The GOG-0213 trial is designed to assess two clinically relevant hypotheses: that bevacizumab plus chemotherapy improves OS (chemotherapy objective) and that secondary surgical cytoreduction in ROC patients improves OS (surgical objective). Nearly 80% of patients in each group received bevacizumab [8]. The median OS was nearly three times longer than expected when the trial was designed. In addition to considering the improvement of clinical care, the selection of bevacizumab may have masked an incremental benefit from surgery. However, the subgroup analysis showed that there was no difference between the surgery group and chemotherapy group in patients receiving bevacizumab (58.5 months vs. 61.7 months; HR = 0.95; 95%CI: 0.65–1.38). The OS of patients without bevacizumab was 32.4 months in the surgery group and 67.0 months in the chemotherapy group, respectively (HR = 2.3, 95%CI: 1.29–4.10). These findings indicated that patients with ROC in the surgery group rather than in the chemotherapy group benefited from bevacizumab. Therefore, the use of bevacizumab might not be the main reason for the difference in PFS and OS estimate between GOG-0213 and the other two trials. Prospective RCTs of surgery combined with bevacizumab will help to answer this question. The results of GOG-0213 should be carefully considered, and chemotherapy alone should not be blindly recommended for all patients to replace SCS + CT.

Second, the criteria for patient selection were different among included studies. Only the SO M 2019 study used the "Tian” model to enroll patients, while the other cohort studies did not use uniform inclusion standard [41]. In GOG-0213 trial, patients were selected on the basis of investigator discretion, without using of objective tools [9]. The DESKTOP-III trial selected patients using the criteria algorithm (AGO score), based on a PS of 0 on the ECOG score, complete resection at primary surgery and ascites of less than 500 ml [11]. The SOC-1 trial selected patients using the iMODEL plus PET-CT scoring algorithm. iMODEL score combined with PET-CT selected more potential candidates than the AGO score [12]. 68% of patients in the DESKTOP-III trial achieved complete resection, similar to the GOG-0213 trial (67%), while 77% of patients in SOC-1 trial achieved complete resection. Therefore, different criteria for patient selection and different maturity of trials may have an impact on the value of surgery.

Finally, the subgroup analysis of RCTs showed that SCS + CT could not bring OS benefit, which was contrary to the results of cohort studies. Cohort study is a retrospective analysis of previous data with a risk of bias, especially selection bias and result reporting bias. Although most included retrospective studies used a propensity score–matched cohort and appropriate statistical techniques to analyze OS, the interpretation of the results should be cautious. More prospective RCTs are still needed to demonstrate whether SCS + CT can bring OS benefits.

In addition, we compared complete resection with chemotherapy alone. Meta-analysis and individual studies clearly showed that complete resection was associated with longer PFS (P < 0.0001) and OS (P = 0.002), and patients who achieved complete resection could benefit from surgery. The DSKTOP III trial reported that the median OS of complete resection was 60.7 months, which was more than 12 months better than the chemotherapy alone group, suggesting that only complete resection could bring OS benefits. Only two studies compared the incomplete resection with chemotherapy alone [12, 36]. The meta-analysis showed that patients with incomplete resection had no significant OS benefit (HR = 1.16, 95% CI: 0.53–2.54, P = 0.71).

Moreover, we compared the effects of complete resection and incomplete resection on survival outcomes. The results of this study showed that complete resection improved PFS (P < 0.001) and OS (P < 0.001). The subgroup analysis of the SOC-1 trial showed that PFS was 19.2 months in the complete resection group and 12.6 months in the incomplete resection group. The subgroup analysis of the DSKTOP III trial showed that the median OS of complete resection was 60.7 months, while the median OS of incomplete resection was only 28.8 months. These findings indicated that the degree of completion of the cell reduction was an important factor affecting the prognosis of patients. Although the meta-analysis included cohort studies, all studies included at least 50 women, and statistical adjustments were used for important prognostic factors. This fact improved the certainty of the estimates.

Only two articles reported chemotherapy toxicity, and there was no statistical difference between the two groups (P > 0.05) [9, 12]. Nine studies reported complications, and 191 of 953 patients had complications within 30 days after surgery. Most patients had grade 1–2 adverse reactions, and only one death related to surgery was reported [9]. Treatment-related adverse reactions usually degrade the quality of life of patients, which is especially important after completing treatment for recurrent cancer, where patients want to enjoy a comfortable standard of living because of the poor prognosis. None of the included articles had quality of life assessment as a component of the studies. Quality of life may be more important for women who have recurrent disease and have significant physical limitations to their life due to the development of the disease and the results of receiving treatment. In this study, the impact of treatment-related adverse events on the life of patients should be considered, which needs more research to confirm. In short, compared with chemotherapy alone, the survival outcome of patients undergoing SCS + CT was much better. In addition, the meta-analysis emphasizes the importance of complete resection for patients, suggesting that the potential benefits of prolonging survival may outweigh the disadvantages of any short-term morbidity associated with surgery.

## Conclusion

In conclusion, SCS-based regimens might result in favorable PFS and OS for patients with ROC. Among patients underwent surgery, only complete resection could bring survival benefits. The incidence of chemotherapy-related toxicity and postoperative complications were low, and serious and fatal adverse reactions rarely occur.

## Supplementary Information

**Additional file 1: Appendix 1.** Search strategies. **Appendix 2**. Risk of bias assessment results for cohort studies using the Newcastle Ottawa Scale. **Appendix 3.**Risk of bias assessment results for cohort studies using the Minors Scale. **Appendix 4.** Risk of bias assessment results for randomized studies using the Jadad Scale. **Appendix 5.** Risk of bias assessment results for randomized studies using Cochrane Risk of Bias Tool. **Table S1**. Baseline characteristics of included patients. **Table S2**. Complications and toxicity. **Figure S1. **Forest plot for included in the patient's baseline analysis. **Figure S2**. Forest plot for OS subgroup analysis of recurrent ovarian cancer. **Figure S3**. Sensitivity analysis of PFS and OS. **Figure S4**. Sensitivity analysis of OS. **Figure S5. **Begg’s and Egger’s test of PFS and OS.

**Additional file 2.**

**Additional file 3.**

**Additional file 4**

**Additional file 5**

**Additional file 6**

## Data Availability

All data is available in this paper.

## Abbreviations

SCS: Secondary cytoreductive surgery
CT: Chemotherapy
ROC: Recurrent ovarian cancer
PFS: Progression-free survival
OS: Overall survival
HR: Hazard ratio
CI: Confidence interval
ITT: Intention-to-treat
PS: Performance status
