# Supplementary material for: The survival outcome and complication of secondary cytoreductive surgery plus chemotherapy in recurrent ovarian cancer: a systematic review and meta-analysis
Source: J Ovarian Res. 2021 Jul 13;14:93. doi: 10.1186/s13048-021-00842-9 (PMC8278673; doi:10.1186/s13048-021-00842-9)
Supplement: Supplementary file 1 — Additional file 1: Appendix 1. Search strategies. Appendix 2. Risk of bias assessment results for cohort studies using the Newcastle Ottawa Scale. Appendix 3.Risk of bias assessment results for cohort studies using the Minors Scale. Appendix 4. Risk of bias assessment results for randomized studies using the Jadad Scale. Appendix 5. Risk of bias assessment results for randomized studies using Cochrane Risk of Bias Tool. Table S1. Baseline characteristics of included patients. Table S2. Complications and toxicity. Figure S1. Forest plot for included in the patient's baseline analysis. Figure S2. Forest plot for OS subgroup analysis of recurrent ovarian cancer. Figure S3. Sensitivity analysis of PFS and OS. Figure S4. Sensitivity analysis of OS. Figure S5. Begg’s and Egger’s test of PFS and OS. [file 13048_2021_842_MOESM1_ESM.docx]

**SUPPLEMENTAL MATERIAL**

**Appendix 1.** Search strategies

**Ovid MEDLINE(R) 1946 to April 01, 2021**

| # | Searches | Results | Type |
| --- | --- | --- | --- |
| 1 | exp Ovarian Neoplasms/ | 86164 | Advanced |
| 2 | (ovar* adj5 (cancer* or neoplas* or carcinom* or tumor* or tumour* or malignan*)).mp. [mp=title, abstract, original title, name of substance word, subject heading word, floating sub-heading word, keyword heading word, organism supplementary concept word, protocol supplementary concept word, rare disease supplementary concept word, unique identifier, synonyms] | 104673 | Advanced |
| 3 | 1 or 2 | 107592 | Advanced |
| 4 | debulk*.mp. | 6439 | Advanced |
| 5 | cytoreduc*.mp. | 9346 | Advanced |
| 6 | 4 or 5 | 14494 | Advanced |
| 7 | exp Surgical Procedures, Operative/ | 3232079 | Advanced |
| 8 | surg*.mp. | 2975892 | Advanced |
| 9 | "surgery".fs. | 2039118 | Advanced |
| 10 | 7 or 8 or 9 | 4365367 | Advanced |
| 11 | “randomized controlled trial”.pt. | 526759 | Advanced |
| 12 | "controlled clinical trial".pt. | 94101 | Advanced |
| 13 | randomly.ab. | 298764 | Advanced |
| 14 | randomized.ab. | 441240 | Advanced |
| 15 | trial.ab. | 464626 | Advanced |
| 16 | groups.ab. | 1843346 | Advanced |
| 17 | exp Cohort Studies/ | 2114576 | Advanced |
| 18 | cohort*.mp. | 644091 | Advanced |
| 19 | 11 or 12 or 13 or 14 or 15 or 16 or 17 or 18 | 4406003 | Advanced |
| 20 | 3 and 6 and 10 | 4672 | Advanced |
| 21 | 19 and 20 | 2574 | Advanced |


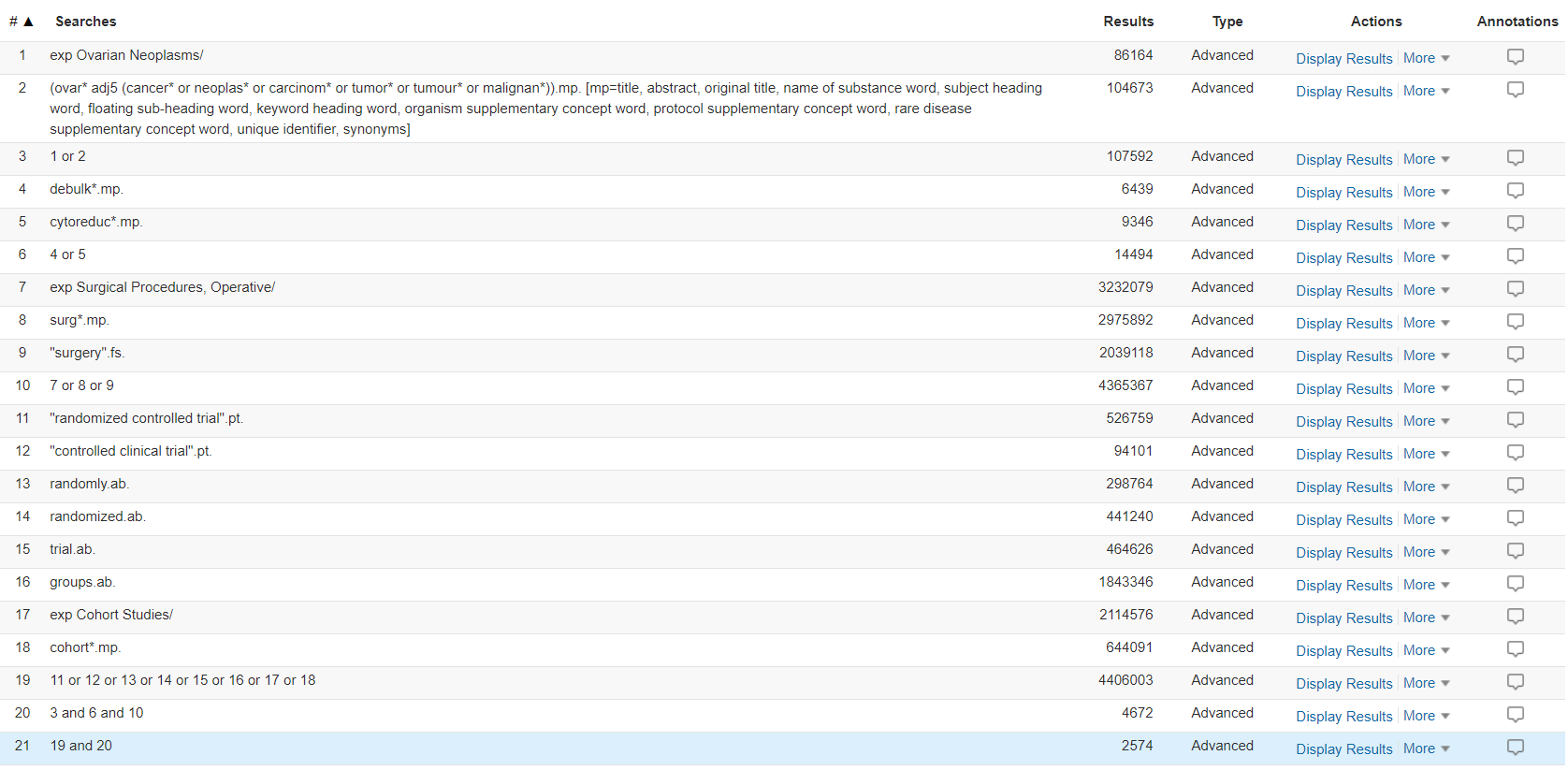


**Embase 1974 to 2021 April 01**

| # | Searches | Results | Type |
| --- | --- | --- | --- |
| 1 | exp ovary tumor/ | 152476 | Advanced |
| 2 | (ovar* adj5 (cancer* or neoplas* or carcinom* or tumor* or tumour* or malignan*)).mp. [mp=title, abstract, heading word, drug trade name, original title, device manufacturer, drug manufacturer, device trade name, keyword, floating subheading word, candidate term word] | 176221 | Advanced |
| 3 | 1 or 2 | 182222 | Advanced |
| 4 | exp surgery/ | 5092379 | Advanced |
| 5 | surg*.mp. | 4341668 | Advanced |
| 6 | 4 or 5 | 6128623 | Advanced |
| 7 | exp controlled clinical trial/ | 855345 | Advanced |
| 8 | random*.mp. | 1913149 | Advanced |
| 9 | trial*.mp. | 2647519 | Advanced |
| 10 | group*.mp. | 5762976 | Advanced |
| 11 | exp cohort analysis/ | 705524 | Advanced |
| 12 | cohort*.mp. | 1260895 | Advanced |
| 13 | series.mp | 860919 | Advanced |
| 14 | 7 or 8 or 9 or 10 or 11 or 12 or 13 | 9269834 | Advanced |
| 15 | debulk*.mp. | 13226 | Advanced |
| 16 | cytoreduc*.mp. | 26241 | Advanced |
| 17 | 15 or 16. | 33777 | Advanced |
| 18 | 6 and 17 | 30519 | Advanced |
| 19 | recurrent.mp. | 578680 | Advanced |
| 20 | recurrence.mp | 637554 | Advanced |
| 21 | 19 or 20 | 1051586 | Advanced |
| 22 | 3 and 14 and 18 and 21 | 1988 | Advanced |


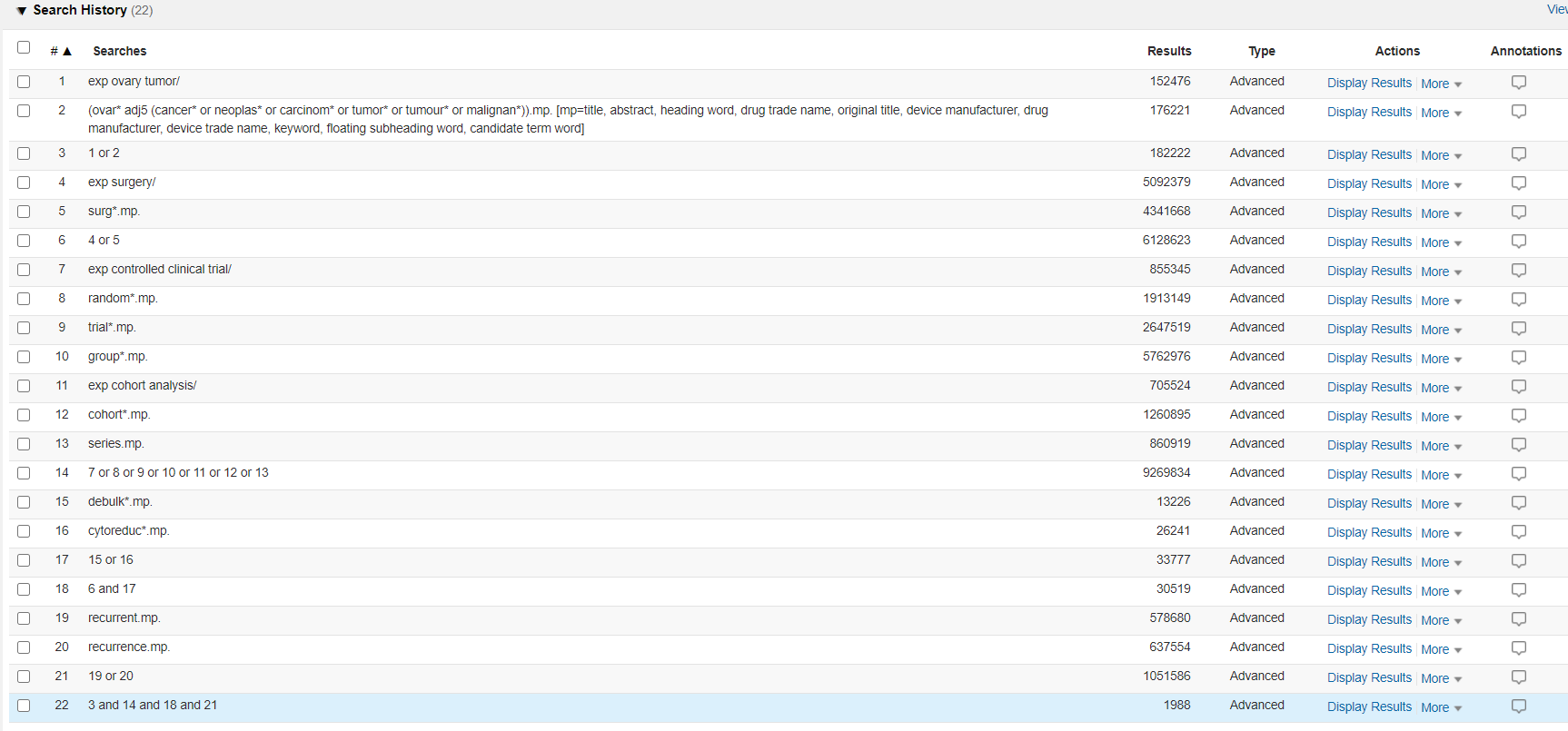


**CENTRAL search strategy**


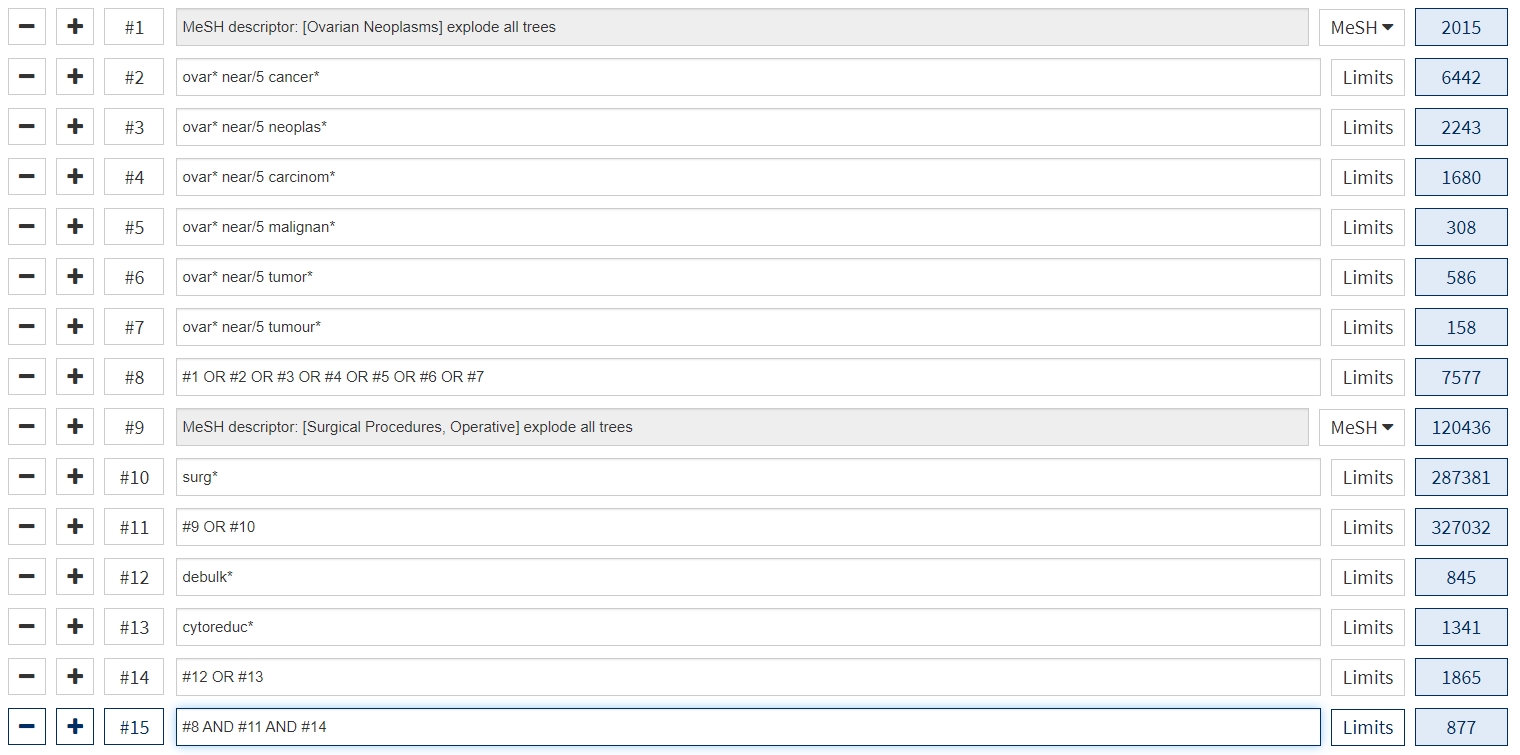


**Appendix 2**. Risk of bias assessment results for cohort studies using the Newcastle Ottawa Scale (>7=low, 4-6=moderate, <3=high)

| Study | Selection | Comparability | Outcome | Total | Risk of bias |
| --- | --- | --- | --- | --- | --- |
| Gockley，2019 | ★★★ | ★ | ★★★ | 7 | Low |
| Felsinger , 2018 | ★★★ | ★ | ★★ | 6 | Moderate |
| Szczesny, 2018 | ★★★ | ★ | ★★ | 6 | Moderate |
| Lee, 2015 | ★★★ | ★ | ★★★ | 7 | Low |
| Oksefjell, 2009 | ★★ | ★ | ★★ | 5 | Moderate |
| Ortega, 2020 | ★★★ | ★ | ★★★ | 7 | Low |
| So M, 2019 | ★★★ | ★ | ★★★ | 7 | Low |
| Güngör, 2005 | ★★★ | ★ | ★★ | 6 | Moderate |
| Takahashi,2017 | ★★★ | ★ | ★★ | 6 | Moderate |
| Kajiyama, 2019 | ★★ | ★ | ★★ | 5 | Moderate |

**Appendix 3.** Risk of bias assessment results for cohort studies using the Minors Scale

|  | Gockley, 2019 | Felsinger, 2018 | Szczesny, 2018 | Lee, 2015 | Oksefjell , 2009 | Ortega, 2020 | So M, 2019 | Güngör, 2005 | Takahashi, 2017 | Kajiyama, 2019 |
| --- | --- | --- | --- | --- | --- | --- | --- | --- | --- | --- |
| The hypothesis/aim/objective of the study was clearly stated | 2 | 2 | 2 | 2 | 2 | 2 | 2 | 2 | 2 | 2 |
| Patients were recruited consecutively | 2 | 1 | 1 | 2 | 1 | 2 | 1 | 1 | 1 | 1 |
| Collection of expected data | 2 | 2 | 2 | 2 | 2 | 2 | 2 | 2 | 2 | 2 |
| Endpoint indicators can appropriately reflect the purpose of the study | 2 | 2 | 2 | 2 | 2 | 2 | 2 | 2 | 2 | 2 |
| Objectivity of endpoint evaluation | 1 | 1 | 1 | 1 | 1 | 1 | 1 | 1 | 1 | 1 |
| Is the follow-up time sufficient? | 2 | 1 | 2 | 2 | 2 | 2 | 2 | 2 | 2 | 2 |
| Lost to follow-up rate is less than 5% | 2 | 2 | 2 | 2 | 1 | 2 | 2 | 2 | 2 | 2 |
| Has the sample size been estimated? | 0 | 0 | 0 | 0 | 0 | 0 | 0 | 0 | 0 | 0 |
| Whether the selection of the control group is appropriate? | 2 | 2 | 1 | 2 | 1 | 2 | 2 | 1 | 2 | 1 |
| Whether the control group is synchronized | 1 | 1 | 1 | 2 | 1 | 1 | 2 | 1 | 1 | 1 |
| Is baseline comparable between groups? | 2 | 2 | 2 | 1 | 1 | 2 | 2 | 1 | 2 | 1 |
| Whether the statistical analysis is appropriate | 2 | 2 | 2 | 2 | 2 | 2 | 2 | 2 | 2 | 2 |
| Total | 20 | 18 | 18 | 20 | 16 | 20 | 20 | 17 | 19 | 17 |
| Risk of bias | Low | Moderate | Moderate | Low | Moderate | Low | Low | Moderate | Moderate | Moderate |

| Study | Randomization | Concealment of allocation | Double blinding | Withdrawals and dropouts | Total | Risk of bias |
| --- | --- | --- | --- | --- | --- | --- |
| Coleman, 2019 | 2 | 2 | 0 | 1 | 5 | Low |
| du Bois,  2020 | 2 | 2 | 0 | 1 | 5 | Low |
| Shi,  2021 | 2 | 2 | 0 | 1 | 5 | Low |

**Appendix 4.** Risk of bias assessment results for randomized studies using the Jadad Scale

**Appendix 5.** Risk of bias assessment results for randomized studies using Cochrane Risk of Bias Tool


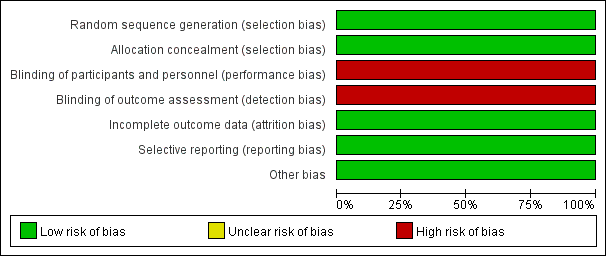


**Table S1**. Baseline characteristics of included patients

| study | PS 0 | FIGO（III or IV） | Histology  (serous) | CA125  (≤105) | absence of ascites | Residual disease | NAC | No. of tumors  (No.≤3) | location of tumors  (intra-abdominal) |
| --- | --- | --- | --- | --- | --- | --- | --- | --- | --- |
| Coleman | | | | | | | | | |
| SCS | - | - | 211 | - | - | - | - | - | - |
| CT | - | - | 207 | - | - | - | - | - | - |
| du Bois | | | | | | | | | |
| SCS | - | - | - | - | - | - | - | - | - |
| CT | - | - | - | - | - | - | - | - | - |
| Shi | | | | | | | | | |
| SCS | 134 | 148 | 158 | 128 | 181 | 83 | 182 | 59 | 136 |
| CT | 144 | 146 | 145 | 115 | 173 | 77 | 173 | 47 | 139 |
| Gockley | | | | | | | | | |
| SCS | 100 | 96 | - | - | 98 | 115 | 10 | - | 146 |
| CT | 335 | 405 | - | - | 278 | 382 | 90 | - | 486 |
| Felsinger | | | | | | | | | |
| SCS |  | 19 | 24 | - | - | 3 | - | 22 | 29 |
| CT |  | 25 | 22 | - | - | 4 | - | 2 | 29 |
| Szczesny | | | | | | | | | |
| SCS |  | 40 | 55 | - | - | - | - | 43 | - |
| CT |  | 200 | 228 | - | - | - | - | 61 | - |
| Lee | | | | | | | | | |
| SCS | 98 | 160 | - | - | - | - | - | - | - |
| CT | 501 | 707 | - | - | - | - | - | - | - |
| Ortega | | | | | | | | | |
| SCS | - | 21 | 25 | - | 36 | - | - | 21 | 21 |
| CT | - | 34 | 32 | - | 30 | - | - | 1 | 31 |
| So M | | | | | | | | | |
| SCS | 22 | 13 | 10 | 18 | 22 | 15 | 8 | 6 | - |
| CT | 28 | 25 | 22 | 24 | 30 | 19 | 14 | 5 | - |
| Güngör | | | | | | | | | |
| SCS | - | - | - | - | - | - | - | 28 | - |
| CT | - | - | - | - | - | - | - | 9 | - |
| Oksefjell | | | | | | | | | |
| SCS | - | 160 | 140 | - | - | 104 | - | - | - |
| CT | - | 506 | 428 | - | - | 202 | - | - | - |
| Takahashi | | | | | | | | | |
| SCS | - | 25 | 26 | 30 | - | - | - | 25 | - |
| CT | - | 62 | 57 | 50 | - | - | - | 39 | - |
| Kajiyama | | | | | | | | | |
| SCS | - | 4 | - | - | - | - | - | - | 15 |
| CT | - | 48 | - | - | - | - | - | - | 93 |
| P value | 0.32 | 0.0001 | 0.43 | 0.07 | 0.44 | 0.24 | 0.76 | 0.0001 | 0.48 |

The number is shown for SCS versus CT. Abbreviations: SCS= secondary cytoreductive surgery; CT= chemotherapy; PS= Performance status; NAC= Neoadjuvant chemotherapy; No.= number.

**Table S2**. Complications and toxicity

|  | Coleman | Shi | Gockley | Felsinger | Ortega | So M | Güngör | Oksefjell | Takahashi | Total |
| --- | --- | --- | --- | --- | --- | --- | --- | --- | --- | --- |
| Intraoperative blood loss | 192 | - | - | 1 | - | - | - | - | - | 193 |
| Bowel resection | 62 | 36 | 59 | 18 | 23 | 20 | 19 | 109 | 1 | 347 |
| Postoperative death | 1 | - | - | - | - | - | - | - | - | 1 |
| Adverse events 30 days after surgery | | | | | | | | | | |
| Pleural effusion | - | 4 | - | 1 | 3 | - | - | - | - | 8 |
| Bowel obstruction | - | 9 | - | 2 | 3 | 4 | 2 | 66 | - | 86 |
| Deep vein thrombosis | 3 | 1 | 2 | - | - | 1 | - | - | - | 7 |
| Reoperation | 20 | - | - | - | 1 | - | 4 | - | - | 7 |
| Infection | - | 18 | - | - | 3 | 1 | 5 | - | - | 27 |
| Cardiac event | - | 4 | - | - | - | - | - | - | - | 4 |
| Other | 17 | 2 | 2 | 3 | - | - | 5 | 0 | 5 | 34 |
| Total N | 40 | 38 | 4 | 6 | 10 | 6 | 16 | 66 | 5 | 191 |
| Chemotoxicity (SCS VS CT) | | | | | | | | | | |
| Total N | 183:194 | 41:31 | - | - | - | - | - | - | - | - |
| P value | P = 0.44 | P> 0.5 | - | - | - | - | - | - | - | - |

Only the data reported in the article is recorded. Abbreviations: SCS= secondary cytoreductive surgery; CT= chemotherapy.


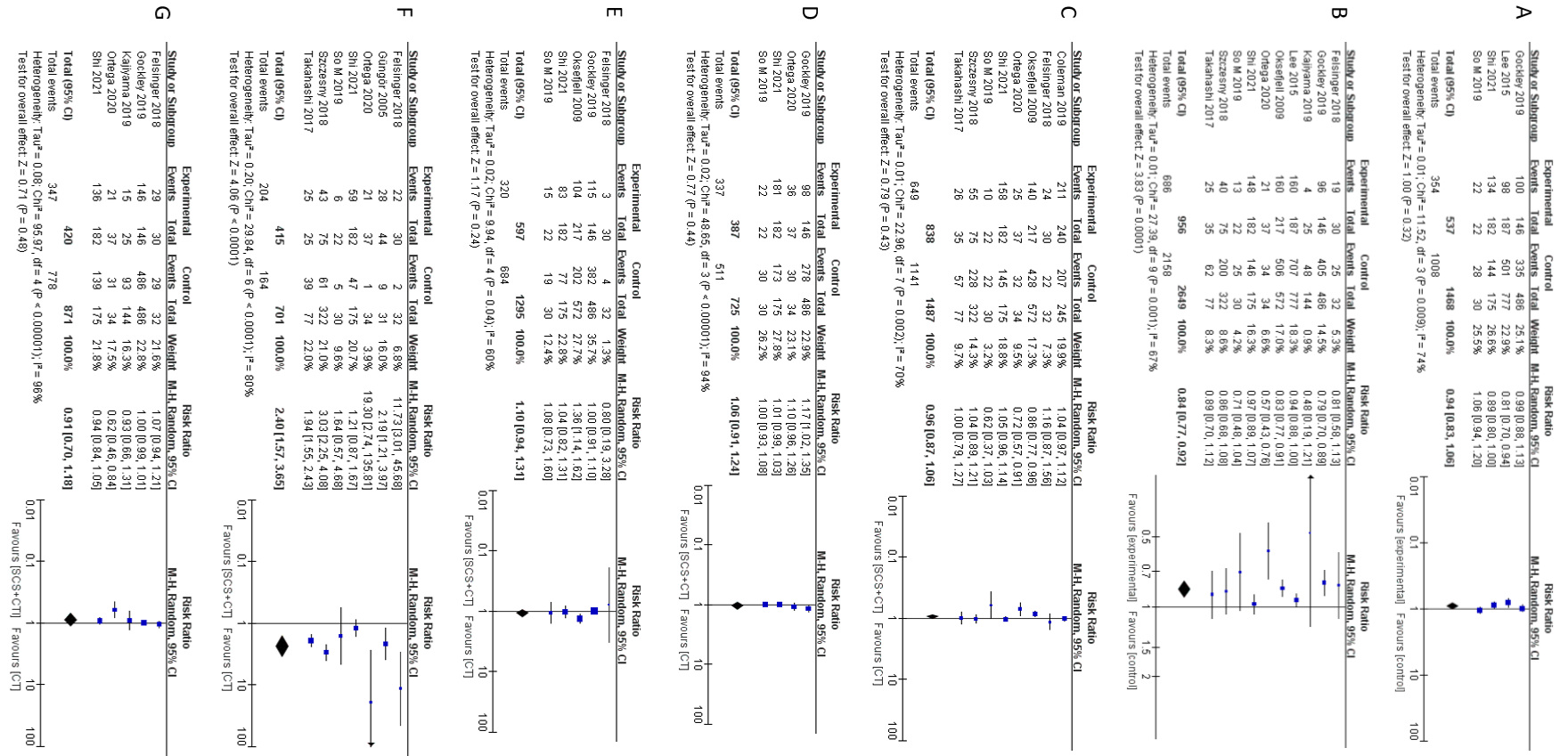


**Figure S1.** Forest plot for included in the patient's baseline analysis. A: ECOG performance status (PS); B: the initial diagnosis of FIGO staging; C: the type of tumor histology; D: ascites; E: residual disease after the initial operation; F: the number of recurring tumors; G: the location of recurrent tumors.


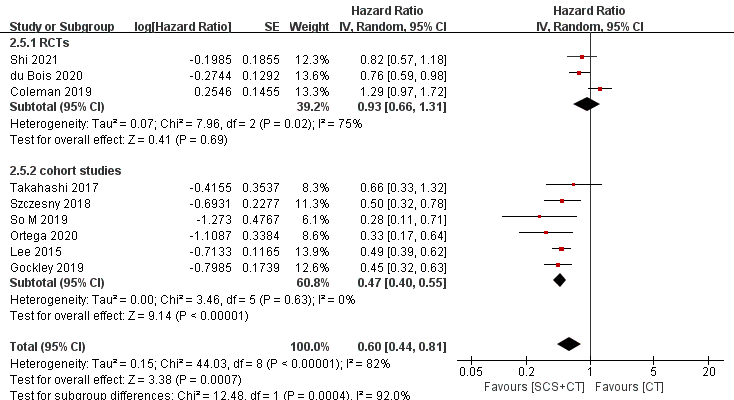


**Figure S2**. Forest plot for OS subgroup analysis of recurrent ovarian cancer. Abbreviations: SCS, secondary cytoreductive surger; CT, chemotherapy.


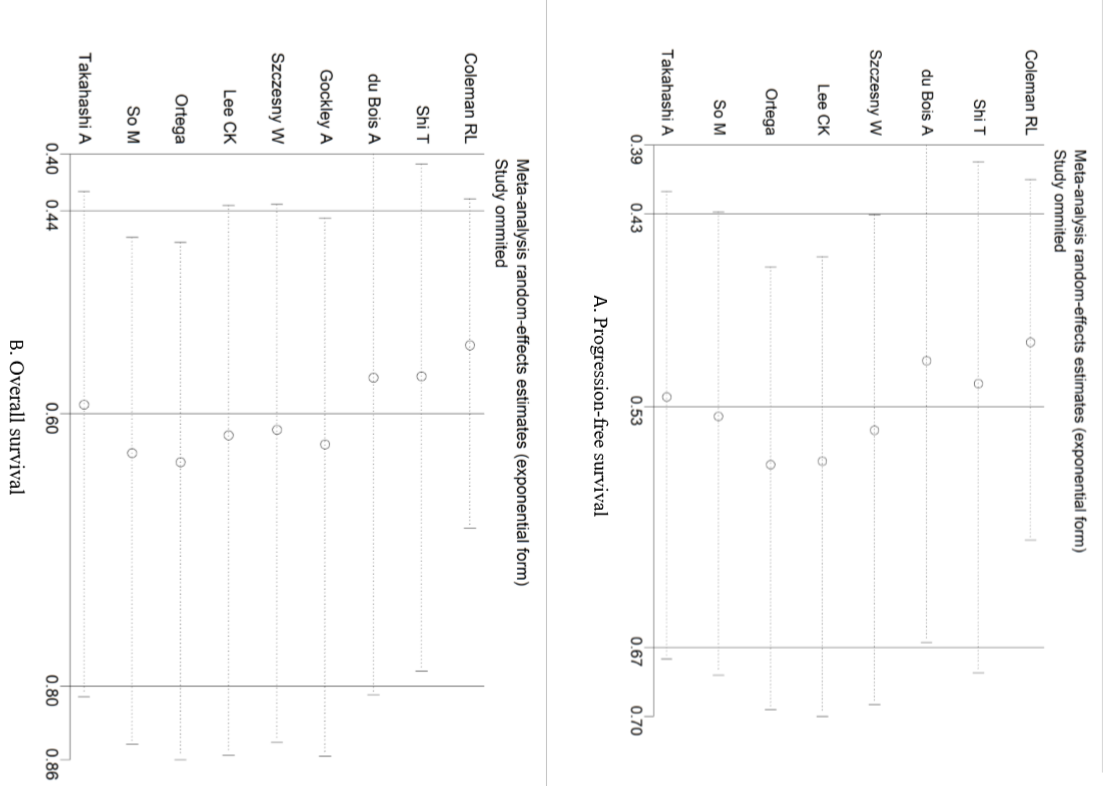


**Figure S3**. Sensitivity analysis of PFS and OS. (Secondary cytoreductive surgery plus chemotherapy versus chemotherapy alone)


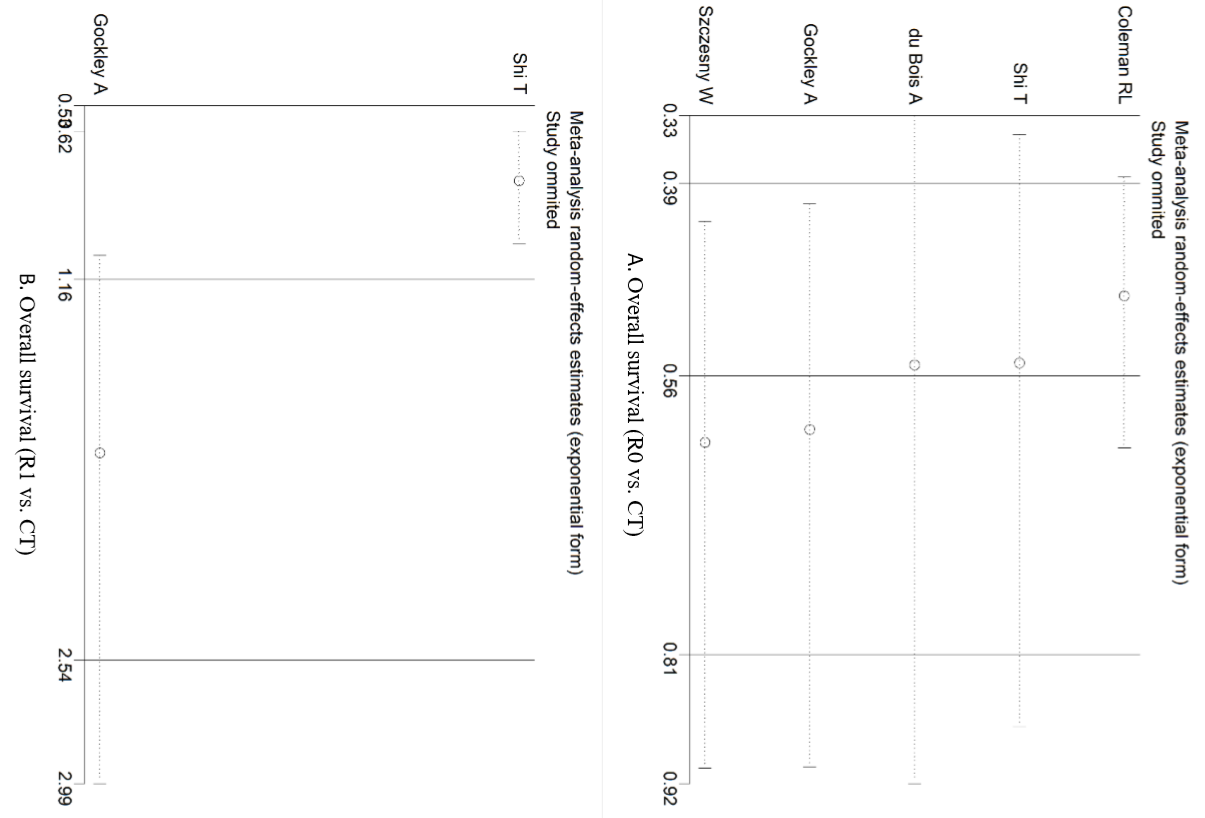


**Figure S4**. Sensitivity analysis of OS. A: complete resection versus chemotherapy alone; B: incomplete resection versus chemotherapy alone.


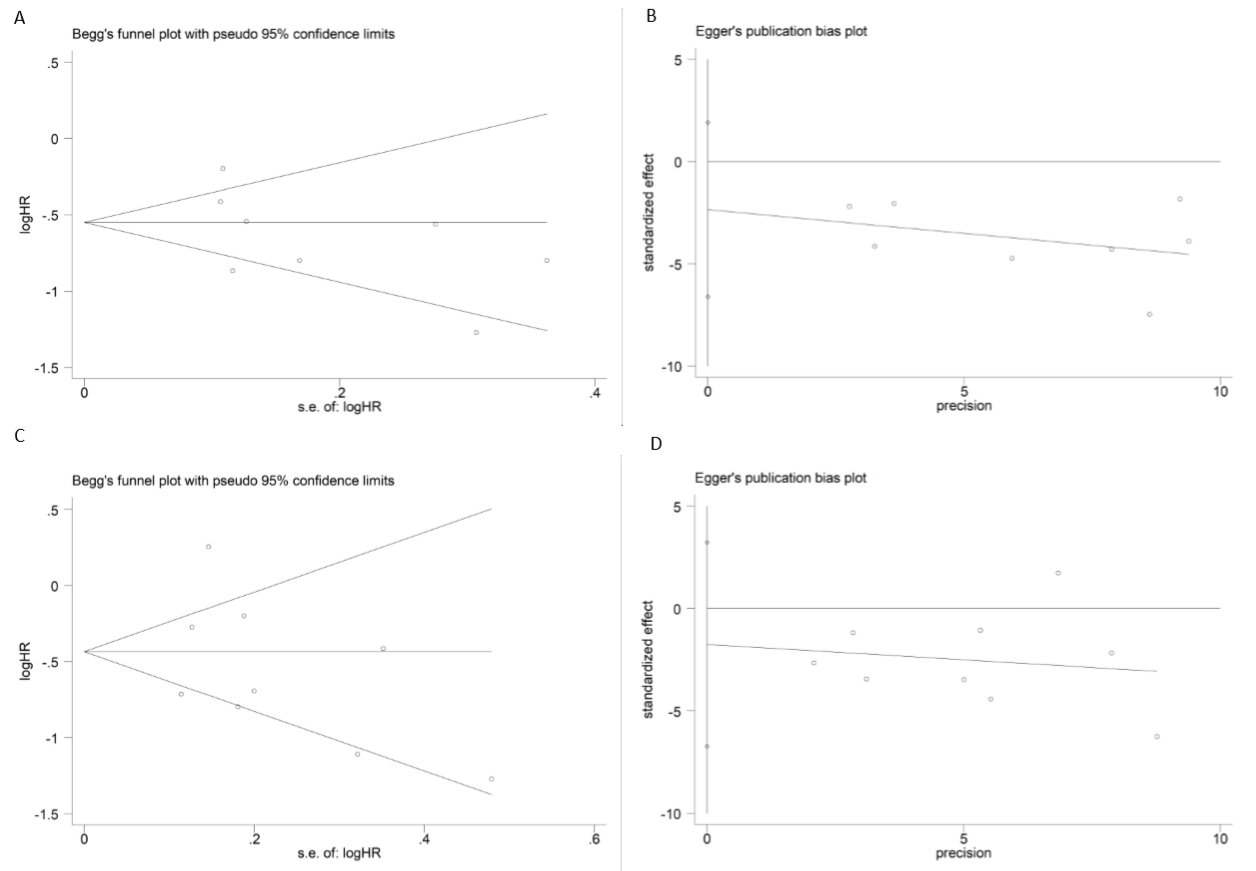


**Figure S5.** Begg’s and Egger’s test of PFS and OS. A: Begg’s test of PFS; B: Egger’s test of PFS; C: Begg’s test of OS; D: Egger’s test of OS.
