# Supplementary figures and images for: The survival outcome and complication of secondary cytoreductive surgery plus chemotherapy in recurrent ovarian cancer: a systematic review and meta-analysis
Source: J Ovarian Res. 2021 Jul 13;14:93. doi: 10.1186/s13048-021-00842-9 (PMC8278673; doi:10.1186/s13048-021-00842-9)

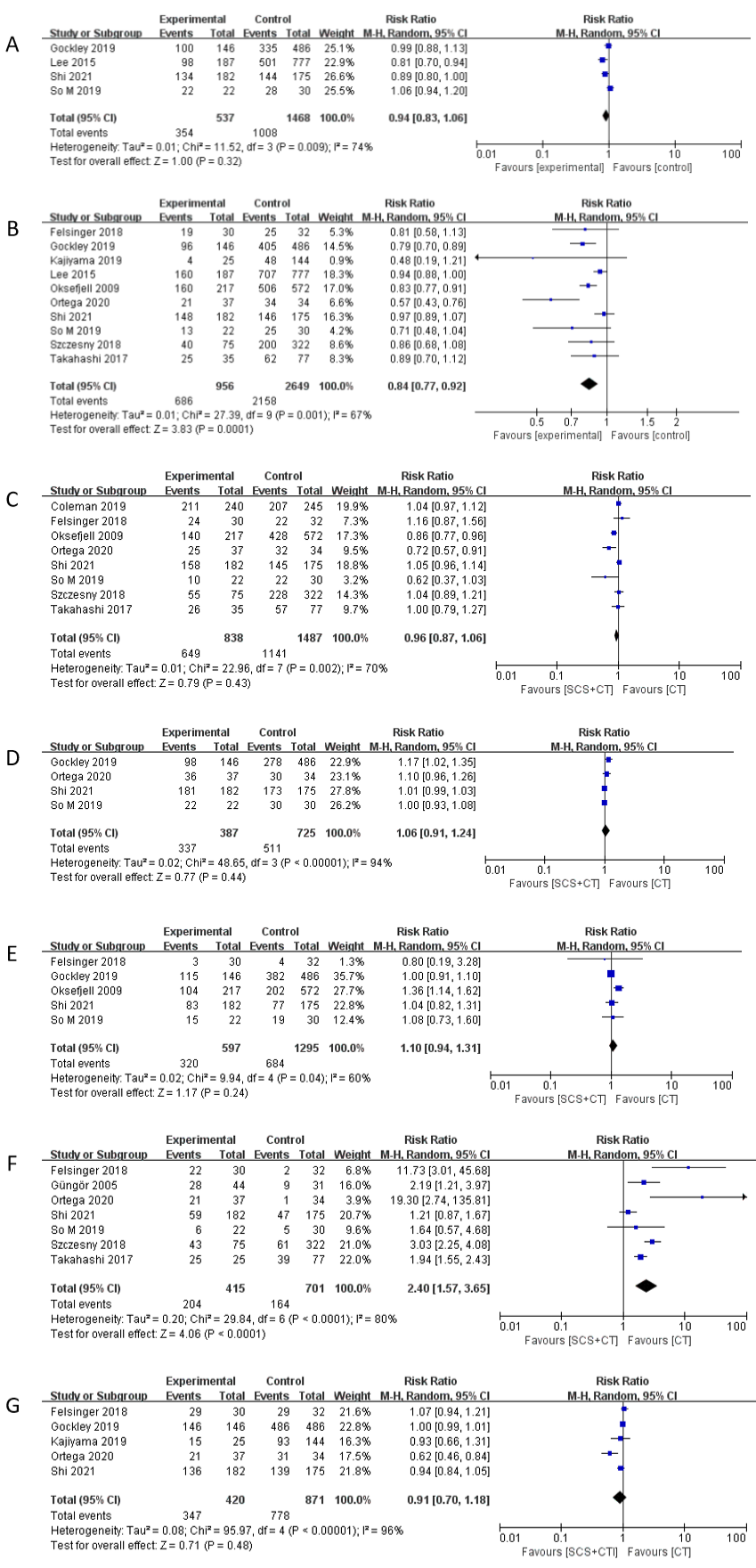

Supplement: Supplementary file 2 — Additional file 2. [file 13048_2021_842_MOESM2_ESM.png]

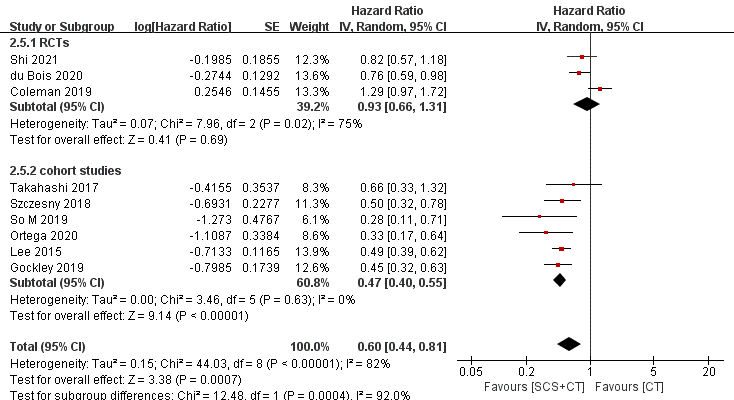

Supplement: Supplementary file 3 — Additional file 3. [file 13048_2021_842_MOESM3_ESM.png]

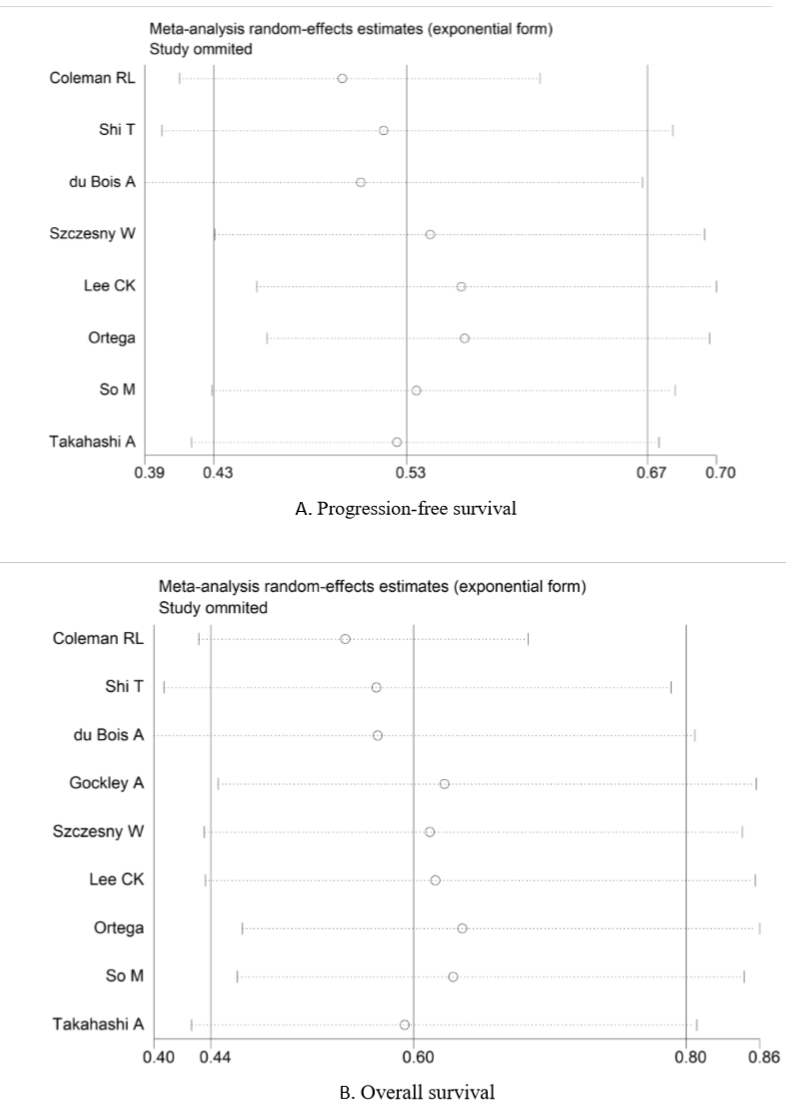

Supplement: Supplementary file 4 — Additional file 4 [file 13048_2021_842_MOESM4_ESM.png]

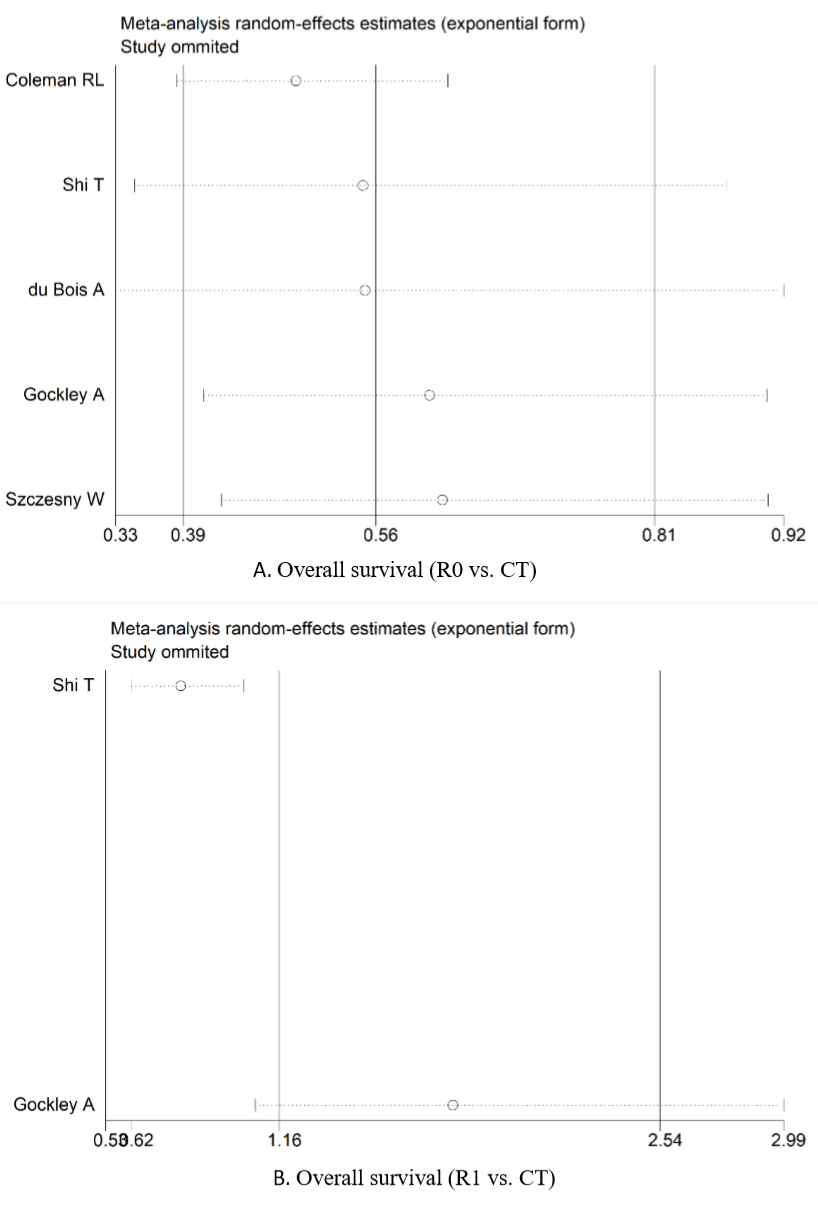

Supplement: Supplementary file 5 — Additional file 5 [file 13048_2021_842_MOESM5_ESM.png]

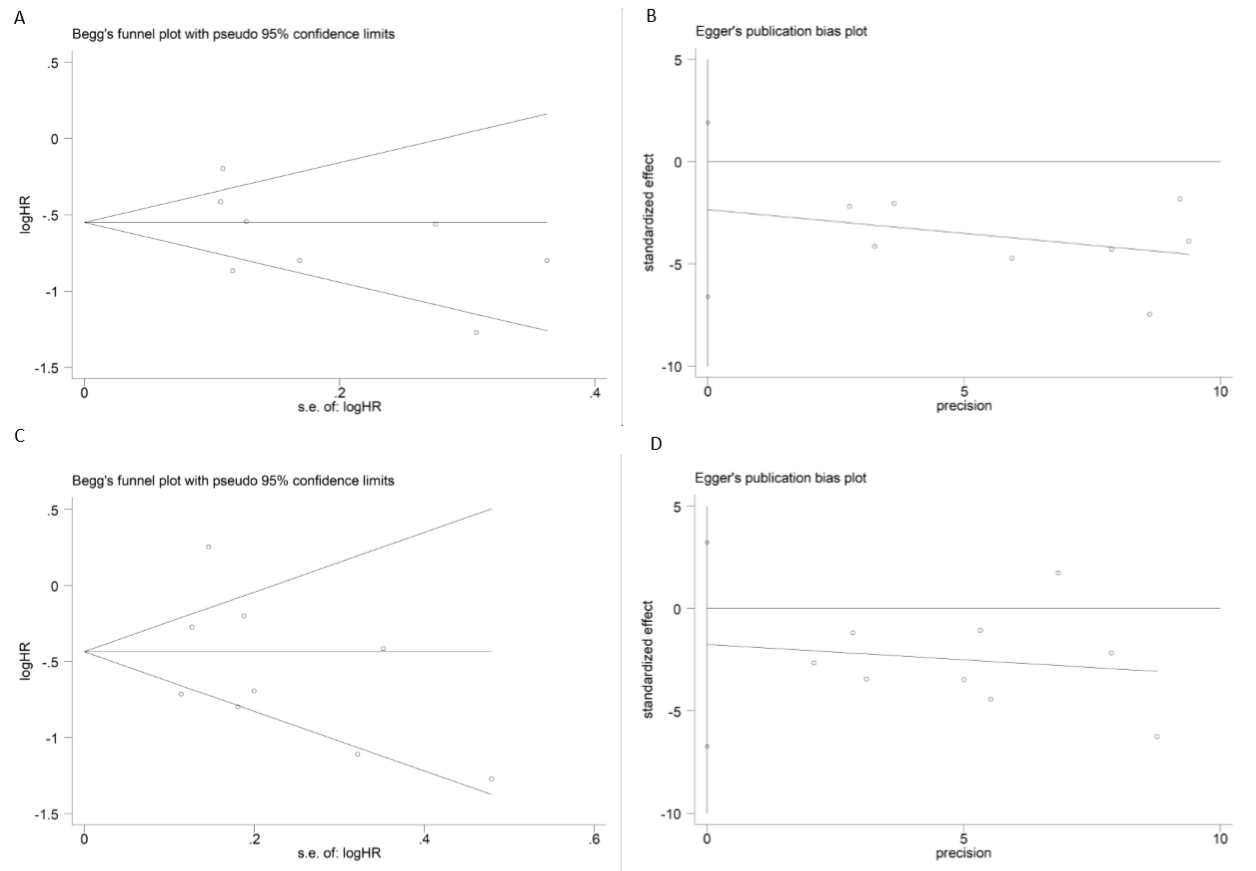

Supplement: Supplementary file 6 — Additional file 6 [file 13048_2021_842_MOESM6_ESM.png]
